# On-Chip Immunoassay for Determination of Urinary Albumin

**DOI:** 10.3390/s91210066

**Published:** 2009-12-10

**Authors:** Wanida Laiwattanapaisal, Temsiri Songjaroen, Thitima Maturos, Tanom Lomas, Assawapong Sappat, Adisorn Tuantranont

**Affiliations:** 1 Centre for Excellence in Omics-Nano Medical Technology Project Development, Department of Clinical Chemistry, Faculty of Allied Health Sciences, Chulalongkorn University, Bangkok, 10330, Thailand; 2 Graduate Programme in Clinical Biochemistry and Molecular Medicine, Faculty of Allied Health Sciences, Chulalongkorn University, Bangkok, 10330, Thailand; E-Mail: stemsiri@yahoo.com; 3 Nanoelectronics and MEMS Laboratory, National Electronics and Computer Technology Centre, Pathum Thani, 12120, Thailand; E-Mails: thitima.maturos@nectec.or.th (T.M.); tanom.lomas@nectec.or.th (T.L.); assawapong.sappat@nectec.or.th (A.S.); adisorn.tuantranont@ectec.or.th (A.T.)

**Keywords:** microalbuminuria, microfluidic system, on-chip immunoassay, poly(dimethylsiloxane)

## Abstract

An immunoassay performed on a portable microfluidic device was evaluated for the determination of urinary albumin. An increase in absorbance at 500 nm resulting from immunoagglutination was monitored directly on the poly(dimethylsiloxane) (PDMS) microchip using a portable miniature fibre-optic spectrometer. A calibration curve was linear up to 10 mg L^–1^ (r^2^ = 0.993), with a detection limit of 0.81 mg L^–1^ (S/N = 3). The proposed system showed good precision, with relative standard deviations (RSDs) of 5.1%, when evaluated with 10 mg L^–1^ albumin (*n* = 10). Determination of urinary albumin with the proposed system gave results highly similar to those determined by the conventional spectrophotometric method using immunoturbidimetric detection (r^2^ = 0.995; *n* = 15).

## 1. Introduction

Detection of albumin contents in the range of 30–300 mg L^–1^ from 24-hr urine samples, known as microalbuminuria, is commonly accepted to be a sensitive forecast of the outcome of nephropathy complications in diabetic patients [1,2]. According to the American Diabetes Association's guidelines, every diabetic patient should be tested annually for microalbuminuria [3]. Because albumin contents in urine are usually low, a sensitive and selective method for an accurate assay is essential.

Several methods have been proposed in the literature for the determination of urinary albumin. Among these, the most routinely used methods are based on immunoassays, due to their high sensitivity and selectivity. These include radioimmunoassay [4], immunoturbidimetry [5], immunonephelometry [6] and enzyme-linked immunosorbant assay (ELISA) [7]. The radioimmunoassay is currently not widely used because of its health hazards, although it is claimed to be a “gold-standard” method [8]. Numerous time-consuming and tedious washing steps are found in the more sophisticated ELISA immunoassay, leading to its disfavour for routine operation. Currently, the immunoturbidimetric method is widely used in most clinical laboratories, and it can be performed in an automated format in parallel with other biochemical tests in the same device. Although significant advantages have been found in the automation of immunoassays, these machines are relatively expensive, usually costing between $50,000 and $200,000 [9]. In addition, these systems require significant laboratory space and are not adaptable to use as portable devices. Furthermore, assay with the conventional instruments is known to consume large amounts of expensive antibody reagent.

Several developments have been explored in an effort to downscale many assay systems, leading to the concept of micro-total analysis systems (μTAS) or “lab-on-a-chip” systems, which are undergoing rapid development. Microfluidic systems are an emerging paradigm of lab-on-a-chip technology, for which several advantages have been demonstrated, including low reagent and sample consumption, fast analysis time and potential portability [10]. Immunoassays are mainly found in clinical analysis, since they benefit from the very high selectivity and affinity of antibody/antigen systems. The tests of latex agglutination reaction are generally simple, cheap, highly specific and do not require specialised skills [11]. In addition, improvements in the performance of the agglutination tests have been continuously attempted to remain competitive with other techniques such as ELISA [9]. Therefore, a miniaturised scale of assay for the latex agglutination immunoassay was deemed worthy of development. The use of microfluidic systems for clinical diagnostic applications has been reviewed extensively in the literature [12–15].

In this work, we demonstrated the potential of a portable microfluidic system for albumin determination that reduced reagent consumption several-fold. The latex agglutination reaction was performed on-chip, and the absorbance changes were simultaneously monitored. Absorbance detection is classified as an unconventional detection method for microfluidic devices due to the short optical path length and the difficulties in coupling the light into and out of microchannels [16]. In an attempt to increase the sensitivity of absorbance detection in a microfluidic chip, a simple custom-made flow cell with a 1-cm path length, similar to a standard cuvette, was fabricated. In our approach, the optical fibres were horizontally aligned at 90° to the fluid flow. Poly(dimethylsiloxane) (PDMS) has become one of the most widely used materials for microfluidic system. PDMS-based microfluidic immunoassays have been previously reported in the literature [17,18]. Attractive for its simple fabrication and low cost, its relatively low refractive index and optical transparency to wavelengths above 230 nm [19,20] have enabled its successful utilisation for on-chip immunoassay and absorbance detection in our system.

## 2. Results and Discussion

### 2.1. Reaction Time

**Figure 1.**
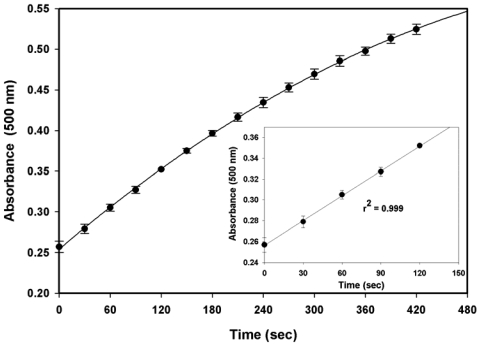
The time course of absorbance change measured at 500 nm by the immunoagglutination reaction. Each point is the mean value from duplicate assays; the error bars represents the standard deviation.

The immunoagglutination reaction, the formation of a complex between albumin and antibody immobilised on latex beads, was proposed for the kinetic detection of urinary albumin. The initial rate of the immunoassay was investigated. Urine samples were diluted 30-fold before analysis, and the protocol described in Section 2.5 was followed. As shown in Figure 1, the agglutination kinetics was noticed to be rather slow, which might be due to the use of surfactant-free latex beads. Surfactants can be utilized to improve the diffusion of particles as well as to enhance antigen–antibody binding [21]. With regard to our experiment, the results demonstrated that a linear response was obtained for an assay time of up to 2 min. It was observed that at longer reaction times a plateau trend appeared. Longer incubation times are not recommended for kinetic immunoassays because they lead to non-specific agglutination and decreased reproducibility [22]. Thus, in our study, the optimal reaction time for each albumin assay was 2 min.

### 2.2. Effect of Carrier-Buffer pH

**Figure 2.**
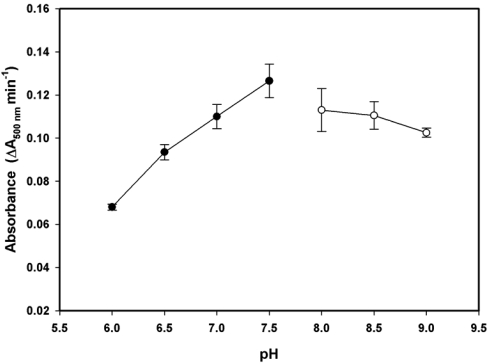
Effect of pH on the rate of immunoagglutination reaction. Solid circles (●), 20 mM phosphate buffer; open circles (○), 20 mM Tris-HCl.

In our experimental design, the streams of latex reagent solution and albumin standard solution or urine sample were introduced to the system simultaneously by using individual injection valves. The carrier buffer delivered both solution streams to the microchip. The pH of the carrier buffer was studied and optimised since dispersion of reagents and buffer can influence the rate of immunoagglutination. The effect of carrier-buffer pH on the rate of the latex agglutination reaction was studied over the range of pH 6–9 with 5 mg L^–1^ albumin. The results, shown in Figure 2, implied that pH 7.5 was the optimum for this assay since the highest signal was obtained at this pH value. Performing the assay at near-neutral pH was also advantageous in terms of the stability of the tubing in the flow system.

### 2.3. Effect of Temperature

**Figure 3.**
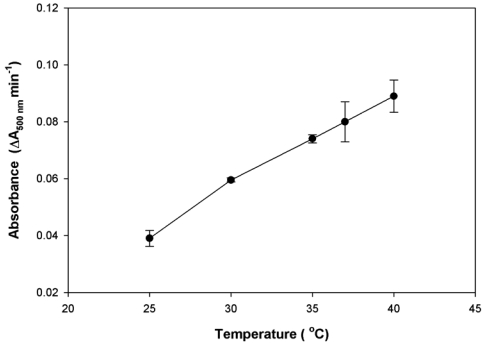
Effect of temperature on the rate of immunoagglutination reaction for determination of urinary albumin.

Temperature is one of the factors affecting the immunoaggregation rate, and its velocity is very sensitive to small temperature changes; therefore, the effect of varying temperature on the assay reaction was studied. The optimised temperature chosen was employed for the microfluidic immunoassay by means of a temperature controller. As shown in Figure 3, it is apparent that the sensitivity of the reaction increased with temperature. Although at 40 °C the highest signal was observed, this high temperature was eliminated in order to avoid protein denaturation. In addition, we observed that air bubbles were easily generated at this temperature. The sensitivity gained from performing the reaction at 37 °C was about 90% of that at 40 °C. Generally, performing the reaction at 37 °C is a common protocol for most immunoassays. For this reason, we kept the temperature constant at 37 °C during all experiments, by means of the temperature controller. However, the reproducibility obtained at both 37 °C and 40 °C was quite poor, which might possibly be due to the flow rate inaccuracies of the pump.

### 2.4. Calibration Curve and Limit of Detection

**Figure 4.**
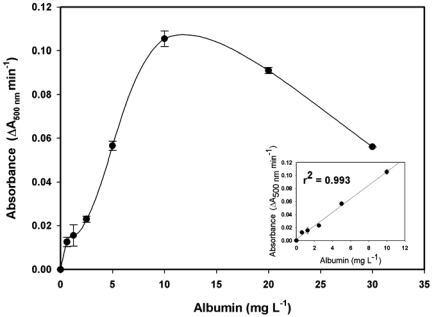
Analytical curve of the microfluidic immunoassay for determining urinary albumin. Inset, a linear range was achieved at 0–10 mg L^–1^ (r^2^ = 0.993).

A 100-μL aliquot of latex reagent solution and 5 μL of a standard solution of albumin were injected into the system via different injection valves. A typical curve for the albumin assay, shown in Figure 4, indicates that at a concentration of albumin higher than 10 mg L^–1^, the absorbance signal dramatically decreased. This could be explained by the “hook effect” or post-zone phenomenon [23], which is attributed to the presence of excess antigen. As shown in the inset, a linear response was obtained in the range of 0 to 10 mg L^–1^ albumin (r^2^ = 0.993). The limit of detection (LOD) was calculated from ten replicate assays of the blank sample. Based on a signal-to-noise ratio (S/N) of 3, the detection limit obtained for albumin was 0.81 mg L^–1^.

Comparing to other microfluidic immunoassays, our method achieved much lower sensitivity than others. For example, Yoon and You [24] described the backscattering particle immunoassays in wire-guide manipulation with achieved detection limit as extremely low as ∼1 pg/mL.

However, our detection limit as low as 0.81 mg L^–1^ was sufficient to detect the urinary albumin, especially in the stage of microalbuminuria. For the early detection of microalbuminuria, a detection limit of 1 mg L^–1^ is desirable. With respect to urinary albumin analysis, our proposed system was slightly more sensitive than such previously described methods as HPLC [25] and the immunoturbidimetric method [26], which were able to obtain detection limits of 6.1 mg L^–1^ and 2 mg L^–1^, respectively.

### 2.5. Interference Effects

To study the effect interfering substances on the determination of albumin, several common interfering substances were added to the human serum albumin standard (5 mg L^–1^). The results, shown in Table 1, indicate that 0.2 mg L^–1^ haemoglobin, 3 mg L^–1^ IgG, 3 mg L^–1^ transferrin, 80 mg L^–1^ bilirubin, and 1,500 mg L^–1^ NaCl each interfered slightly in the system, as recovery ranges from 110–115.3% were obtained.

These slight interferences could be explained by cross-reaction of the antibody with other proteins, especially in high-concentration conditions. These findings confirm observations reported previously in the literature [22]. At high concentrations, the colour of bilirubin is known to disturb the absorbance measurement and generate noise signals. Moreover, this immunoassay requires optimal ionic strength; therefore, a high concentration of salt might disturb the agglutination reaction [23]. With our system, 200 mg L^–1^ ascorbic acid and 1,250 mg L^–1^ glucose were not found to significantly interfere with the experimental system (recovery 100–101.3%).

### 2.6. Precision and Carry-Over Affect

One advantage of our proposed microfluidic system is reusability of the microchip. Because a known property of PDMS is that it can easily adsorb proteins during assay, the carry-over effect was evaluated. High (10 mg L^–1^) and low (1 mg L^–1^) albumin concentrations were injected into the system. The formula (b1–b3)/(a2–b3) × 100 was used for calculation of the percentage of carry-over [27], where a and b represent the absorbances obtained at the high and low albumin concentration injections, respectively, and their numbers represent the order of injection. With our system, carry-over was calculated to be 3.75%, which is acceptable for the current system. The repeatability and precision of the system were also assessed. The same-day precision was obtained at 5.1% coefficient of variation (CVs) when evaluated with a 10 mg L^–1^ albumin standard (*n* = 10). The results indicated that the microchip can be reusable with good repeatability.

### 2.7. Analysis of Urine Samples

**Figure 5.**
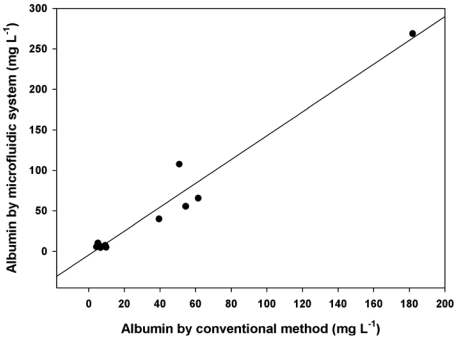
A scatter-plot of results determined by the microfluidic system and the conventional method for the urinary albumin. The regression analysis relation is y = 0.993(±0.04)x + 0.6898(±2.9); *n* =15, r^2^ = 0.995.

To evaluate the accuracy of the proposed system for clinical sample analysis, urine samples were assayed, and the results were compared with those obtained from the conventional immunoturbidimetric method. Urine samples (*n* = 15) were diluted with normal saline 15-30-fold before assay. A scatter-plot of the results obtained by both methods is shown in Figure 5 (r^2^ = 0.995, *n* = 15). The regression analysis obtained the linear relation y = 0.993(±0.04)x + 0.6898(±2.9).

**Figure 6.**
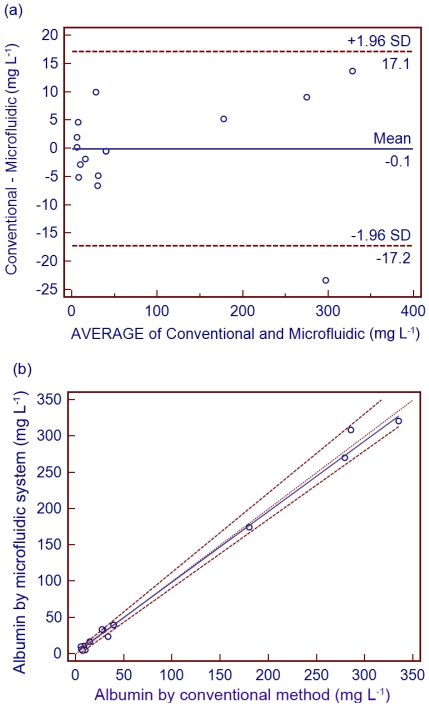
Comparison of results from the microfluidic system and the conventional immunoturbidimetric method for the urinary albumin assay. Results shown with (a) Bland-Altman bias plot and (b) Passing-Bablok regression analysis.

A Bland-Altman plot [28] comparing the microfluidic system with the conventional immunoturbidity method is displayed in Figure 6a.

The results demonstrated that there is no apparent bias for urinary albumin determined by the proposed system because the differences between the two methods are entirely within the mean ± 1.96 SD. A Passing-Bablok regression [29] was employed to assess the agreement between both methods, as shown in Figure 6b. The equation for the Passing–Bablok regression line, y = 1.0396x + 1.1594, is displayed. Within a 95% confidence interval, the results indicated that our proposed microfluidic system for determination of urinary albumin was in good agreement with the conventional immunoturbidimetric method. A linearity test indicated no significant deviation (*p* > 0.1).

## 3. Experimental Section

### 3.1. Chemicals, Reagents and Samples

All chemicals were of analytical reagent grade. Haemoglobin, IgG, transferrin, ascorbic acid, D-(+)-glucose, bovine and human serum albumin were obtained from Sigma (St. Louis, MO, USA). Bilirubin was purchased from Fluka (Buchs, Switzerland). NaCl, potassium dihydrogen phosphate (KH_2_PO_4_), and dipotassium phosphate (K_2_HPO_4_) were products from Merck (Darmstadt, Germany). Poly(dimethylsiloxane) (PDMS, Sylgard 184) and its curing agent were obtained from Dow Corning (Midland, MI, USA). Photoresist (SU-8 2100) and developer were purchased from MicroChem (Newton, MA, USA). All solutions were prepared in Milli-Q water. Regarding the reagents used for the latex agglutination test, the surfactant-free plain polystyrene latex beads with 50–70 nm bead size were purchased from BioSystems (Barcelona, Spain). The latex reagent was freshly prepared according to the manufacturer's instructions. The antibody-coated latex beads in borate buffer were freshly prepared by mixing them together in ratio of 1:1, and then the solution was further diluted twofold with 20 mM phosphate buffer (pH 7.5), corresponding to OD_500 nm_ of 0.5 and a protein concentration of 2.64 mg mL^–1^. The microalbumin assay kit used for method validation was supplied by Randox Laboratories (Antrim, United Kingdom). Anonymous human urine samples were collected from Chulalongkorn General Hospital and Rajavithi Hospital, Bangkok, Thailand. The urine specimens were centrifuged at 1,500 rpm (214 g) for 5 min before subjecting the supernatant to assay with the microfluidic system.

### 3.2. Apparatus and Instrumentation

A miniature fibre-optic spectrometer (USB4000) with an LS-1-LL tungsten light source was a product of Ocean Optics Inc. (Dunedin, FL, USA). The two-channel syringe pump (Fusion 200) was a product of Chemyx (Stafford, TX, USA). Injection valves (V-451), PTFE tubing with a 0.5-mm i.d. and all PEEK connectors were products of Upchurch Scientific (Oak Harbor, WA, USA). Tygon tubing was obtained from Bio-Rad Laboratories (Richmond, CA, USA).

A spin coater (model WS-400A-6NPP, Laurell technologies Corp., North Wales, PA, USA) was used for spin coating of the photoresist onto the silicon substrate for mould fabrication. The UV-lithography process was done using an MJB4 mask aligner (SUSS microtec, Germany). The oxygen-plasma cleaner (PDC-32G) used for PDMS surface oxidation prior to bonding was a product of Harrick Scientific Corp. (Ossining, NY USA). The temperature-control system, set at 37 °C with ±0.2 °C accuracy, was made locally in our laboratory. A UV-VIS spectrophotometer (Evolution 600, Thermo Scientific, USA) was used for determination of urinary albumin based on the conventional immunoturbidity for comparison to our method.

### 3.3. PDMS Microchip Fabrication and Design

The masks used for UV-lithography were designed using the L-edit program (version Pro v8.03) and then printed onto a transparency film. The microchip comprised three main areas: a y-shaped microchannel, a mixing zone and a detection zone. The dimensions of the whole microchannel were 500 μm wide and 100 μm deep. The silicon wafer was coated with a 100-μm-thick photoresist (SU-8) by a spin-coating technique. To cast the PDMS microchip, the prepolymer was prepared by mixing the curing agent with PDMS prepolymer at a 1:10 weight ratio, and then the PDMS mixture was degassed and poured over the SU-8 master mould.

Two PDMS sheets were separately prepared for the upper and lower layers. The upper layer, a 2-mm thick PDMS cast containing the channel structure, was peeled off from the master, and holes for inlets were drilled into the PDMS chip using metal pipes (1.5 mm i.d.). A modified flow cell made from a polystyrene cuvette with a 1-cm path length and a total volume of 90 μL was incorporated into the lower, 6-mm thick PDMS layer. A hole was punched above the detection zone of the lower microchip for receiving the fluid flow from the upper PDMS layer. This type of microchip was successfully developed for determination of urinary creatinine in the previous report by our group [30].

Before assembling the complete microchip, both upper and lower PDMS slabs were exposed to oxygen plasma and then immediately bonded together. Subsequently, the microchip was further heated on a hotplate at 70 °C for 10 min to strengthen the bonding. Tygon tubes (0.8 mm i.d.) were connected to the microchip for inlets and outlets and glued with epoxy resin. Finally, the microchip was filled with bovine serum albumin (1 mg mL^–1^) and held overnight in order to reduce non-specific binding and improve hydrophilicity of the chip.

### 3.4. Microfluidic System Set-Up

**Figure 7.**
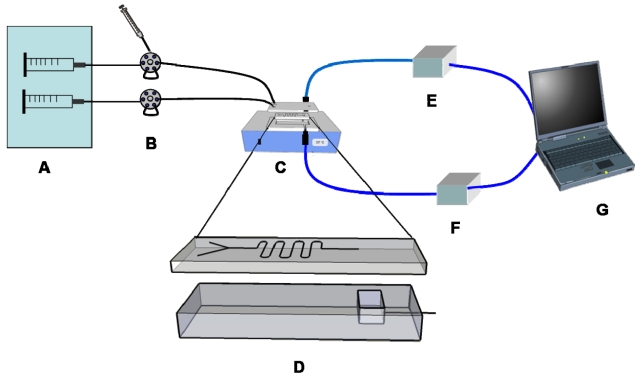
Microfluidic system set-up for on-chip immunoassay and absorbance detection. (A) dual syringe pump; (B) injection valves; (C) temperature controller with PDMS microchip inside; (D) detailed view of the PDMS microchip; (E) miniature spectrometer; (F) light source; (G) portable computer.

The microfluidic system components for the on-chip immunoassay are shown in Figure 7. A two-channel syringe pump (0.098 μm/step, CVs of flow rate accuracy <1%) equipped with two 20-mL plastic syringes (Terumo®, Terumo Corporation, Tokyo, Japan) was used to deliver the carrier buffer (20 mM phosphate, pH 7.5). Two injection valves, with sample injection loops of 100 μL and 5 μL, were used for introduction of the latex reagent and urine sample, respectively. The PDMS microchip was placed in a temperature controller which maintained a constant temperature of 37 °C ± 0.2 °C throughout the assay. The absorbance detection was performed on-chip using the fibre-optic cables, which were connected to a USB4000 spectrometer and tungsten light source. The cables were horizontally arranged in the detection zone of the PDMS microchip at 90° to the fluid flow. Absorbance and spectral changes were recorded using the software provided by the manufacturer of the spectrometer, which was controlled by a portable computer. In terms of system set-up *i.e.*, utilizing a microchip with PDMS Y-channel and USB4000 fibers optic spectrometer for latex immunoagglutination assay, our proposed system was similar to the systems described by Lucas *et al.* [13], Han *et al.* [31], and Heinze *et al.* [32]. Nevertheless, the apparent difference was the detection technique as we utilized a simple absorbance detection rather than a light scattering assay. Light scattering optical systems are more difficult to construct whereas turbidimetry is more broadly applicable [33].

### 3.5. Assay Procedure

Prior to the experiments, the temperature controller was turned on, and the buffer was flowed through the microchip until stable signals were observed. Unless otherwise stated, the buffer used was 20 mM phosphate buffer, pH 7.5, and the flow rate was fixed at 40 μL min^–1^. A 100-μL aliquot of latex reagent solution and 5 μL of diluted urine sample or human serum albumin standard were injected into the system via the separate injection valves. After the stream of mixed solutions reached the detection zone, the flow was allowed to stop. Increments in absorbance of 500 nm due to the immunoagglutination reaction were recorded for 2 min at 30-sec intervals. To regenerate the surface of the microchip for another round of injection, the pump was turned on and buffer circulated until the baseline was gradually reduced to the original signal. Including the washing step, each assay run required about 10 min.

### 3.6. Immunoturbidimetric Method

The proposed method was validated for accuracy by comparison to the immunoturbidimetric method using the commercial microalbumin kit from Randox Laboratories. A UV-VIS spectrophotometer set at 340 nm was used according to the manufacturer's instructions. First, 1.0 mL of reagent buffer was mixed well with 0.1 mL of the sample/standard, and the initial absorbance (A1) was measured against water. Subsequently, 0.1 mL of the antibody solution was added. After thorough mixing, the solution was incubated at room temperature for 30 min. The solution was mixed again, and then the final absorbance (A2) of each solution was measured. The unknown concentrations of the samples were interpolated from the semi-log standard curve, which was plotted between the albumin concentrations and the change in absorbance (A2-A1) using the microalbumin calibration series supplied with the kit. Quality control of the assay was performed using the two levels of control samples provided with the kit.

## 4. Conclusions

An on-chip immunoassay for the determination of low levels of urinary albumin was proposed. Universal absorbance detection was utilised for monitoring the immunoagglutination reaction by means of a portable miniature fibre-optic spectrometer. The limitation of a short path length in the microfluidic device was overcome by utilising a custom-made flow cell; together, this approach considerably improved the assay sensitivity. With our proposed system, the reagents can be used more economically than in the conventional immunoassay, allowing for a tenfold lower reagent consumption. Other advantages of the presented system are simplicity of operation and convenience, low cost of analysis, good reproducibility of results, reusability of the microchip and full portability for in-field analysis.

## Figures and Tables

**Table 1.** Effects of the tested substances on the determination of urinary albumin using the proposed microfluidic system.

**Tested substances**	**Concentration (mg L^–1^)**	**%Recovery**
albumin	5.0	100.0 ± 3.7
haemoglobin	0.2	110.0 ± 2.8
IgG	3.0	111.3 ± 0.9
transferrin	3.0	115.3 ± 2.8
bilirubin	80.0	114.0 ± 0.9
NaCl	1,500.0	110.7 ± 1.8
ascorbic acid	200.0	101.3 ± 3.7
glucose	1,250.0	100.0 ± 0.0
